# What is new in critical care echocardiography?

**DOI:** 10.1186/s13054-018-1970-8

**Published:** 2018-02-22

**Authors:** Philippe Vignon

**Affiliations:** 1 Medical-surgical intensive care unit, Dupuytren Teaching hospital, Limoges, France; 2 INSERM Clinical Investigation Center 1435, Dupuytren Teaching hospital, Limoges, France; 3 University of Limoges, Limoges, France

Critical care echocardiography (CCE) is performed and interpreted by the intensivist at the bedside to help in the diagnostic work-up and to guide therapy for patients with acute circulatory and respiratory failure. Due to the statement of educational standards, the validation of echocardiography against alternative hemodynamic monitoring tools, and continuous technological improvement, CCE is increasingly being considered as an unparalleled technique to hemodynamically assess critically ill patients. Extremely active research in the field of CCE has resulted in recent improvements which warrant comment (Table 1).

**Table 1:** Main recent proceedings in the use of CCE

New developments in echocardiography in the critically ill
1. CCE is currently recommended as the first-line modality to assess patients with shock
2. CEE relies on basic and advanced levels of competence corresponding to distinct standardized educational requirements
3. CCE guides the front-line intensivist in assessing fluid requirement
4. CCE is currently recommended for the diagnosis of acute respiratory distress syndrome
5. CCE uses a simple yet robust and sound diagnostic approach
6. CCE becomes a monitoring rather than a diagnostic tool
7. CCE benefits from continuous technological refinements (e.g., speckle tracking)
8. CCE is ideally suited for guiding tailored management of unstable patients

*CCE* critical care echocardiography

CCE provides unique morphological and functional information in real time to depict the hemodynamic profile of shock [1, 2]. The recent consensus on circulatory shock and hemodynamic monitoring of the European Society of Intensive Care Medicine suggests that CCE is the preferred modality to initially evaluate the type of shock as opposed to more invasive techniques. To provide adequate training of intensivists, international roundtables have recently detailed the theoretical knowledge and practical skills that should be mastered to achieve competence in both basic and advanced CCE. Basic CCE should be part of the initial training of all intensivists, whereas advanced CCE is an optional component of competency since it requires a more extensive and specific training [3]. Computerized simulation accelerates the learning curve of transesophageal echocardiography for the hemodynamic assessment of critically ill patients [4].

CCE helps to assess the fluid responsiveness and both the efficacy and tolerance of blood volume expansion in patients with shock, irrespective of their respiratory status [5]. A recent multicenter study prospectively assessed 540 ventilated patients with shock of any origin using transesophageal echocardiography and systematic passive leg raise to mimic a transient central blood volume expansion [6]. With the exception of the superior vena cava collapsibility index which was obtained in nearly all patients, dynamic indices proposed to predict fluid responsiveness were obtained in approximately 78% of patients. Respiratory variation of left ventricular outflow tract Doppler maximal velocity was the most sensitive index, whereas the superior vena cava collapsibility index was the most specific to predict fluid responsiveness. Due to a large range of uninformative values of dynamic indices, authors proposed using high yet specific threshold values to accurately predict fluid responsiveness in patients for whom inefficient fluid loading would be detrimental (e.g., acute respiratory distress syndrome), whereas a more liberal approach could be used in the remaining patients with repeated CCE assessment of both efficacy and tolerance of serial mini-fluid loadings [6]. Importantly, CCE solely can ascribe the respiratory variation of pulse pressure or stroke volume (pulse contour method) to a right ventricular failure (Fig. 1), and accordingly avoid a false-positive diagnosis of fluid responsiveness [7].

**Fig. 1:**
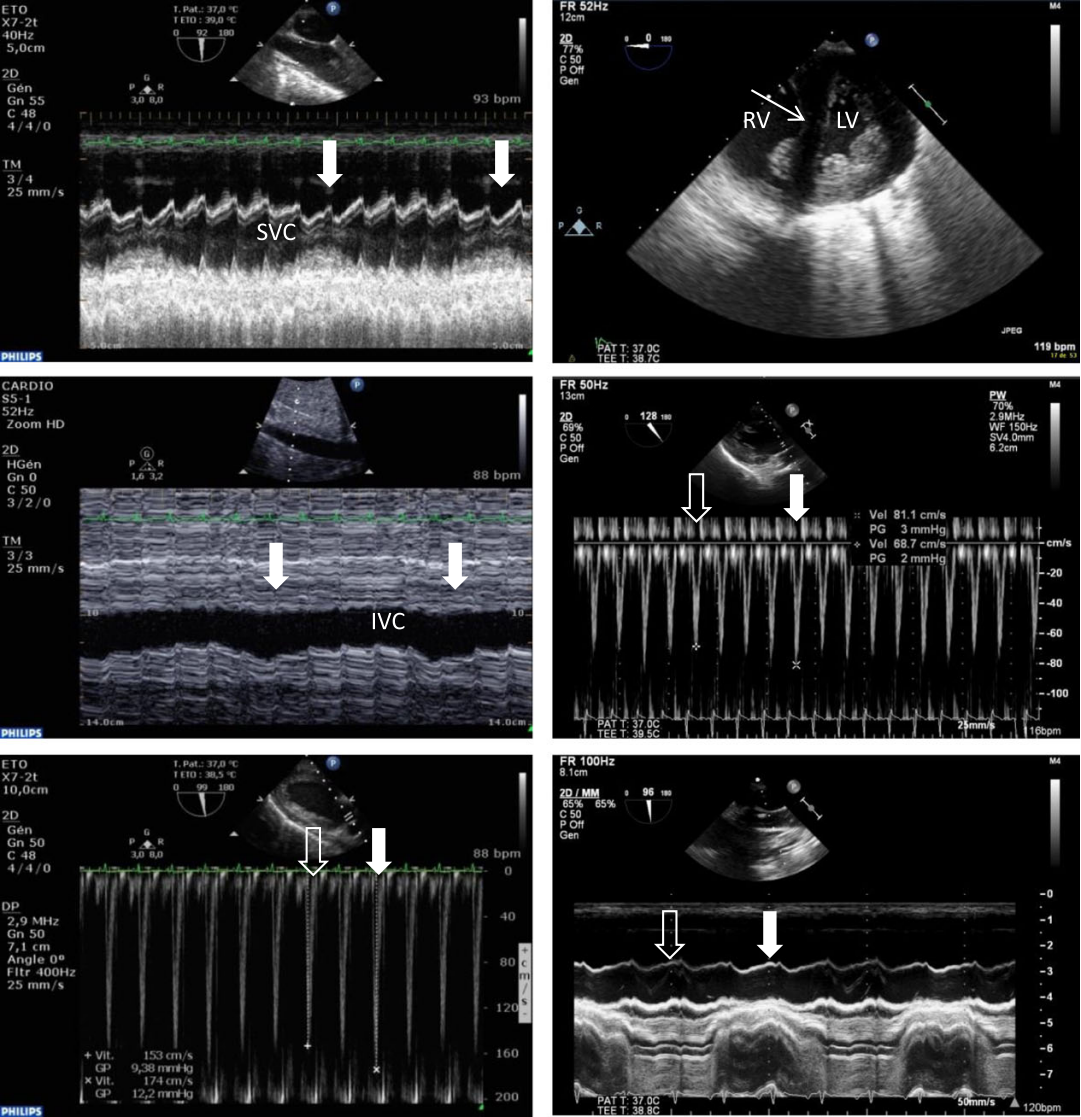
Hemodynamic assessment using transesophageal echocardiography in two patients presenting with shock of distinct origin. In the first patient (left panels), inadequate cardiac output was ascribed to a persisting preload dependence, as reflected by concordant and significant respiratory variations (closed thick white arrow: mechanical insufflation) of the superior vena cava (SVC) diameter (upper left, collapsibility index), of the inferior vena cava (IVC; middle left, distensibility index), and of maximal Doppler velocity recorded in the left ventricular outflow tract (lower left, open thick arrow: expiration phase). In this patient, cardiac output significantly increased after fluid loading. In the second patient who was under protective mechanical ventilation for an acute respiratory distress syndrome (right panels) and exhibited tissue hypoperfusion, a 15% pulse pressure variation suggested fluid responsiveness. Transesophageal echocardiography disclosed an enlarged right ventricle (RV) in conjunction with a paradoxical septal motion consistent with an acute cor pulmonale (upper right, thin arrow) and significant respiratory variations of maximal Doppler velocity recorded in the left ventricular outflow tract (middle right, open thick arrow: expiration phase; closed thick arrow: mechanical insufflation). Nevertheless, the absence of any respiratory variations of the superior vena cava diameter suggested the absence of fluid responsiveness, but rather a systemic venous congestion associated with acute cor pulmonale (lower right). Accordingly, both pulse pressure variation and respiratory variations of maximal Doppler velocity recorded in the left ventricular outflow tract were interpreted as false-positive results. Blood volume expansion was considered potentially inefficient and detrimental, ventilator settings were modified, and inhaled nitric oxide was initiated to further unload the right ventricle. LV left ventricle

CCE plays a major role in the diagnostic work-up of patients presenting with acute respiratory failure [8]. In assessing left ventricular diastolic properties and filling pressure, CCE accurately identifies cardiogenic edema or weaning pulmonary edema, and potentially associated cardiopathy. According to the Berlin definition of the acute respiratory distress syndrome, echocardiography is recommended to exclude hydrostatic pulmonary edema. Since the prevalence of acute cor pulmonale in this clinical setting reaches 22%, a risk score has been proposed to select patients who should undergo CCE [9]. In addition, transesophageal echocardiography is the reference diagnostic modality to identify potentially associated patent foramen ovale [8].

Used as either a focused cardiac evaluation at the basic level or a comprehensive hemodynamic assessment modality at the advanced level, CCE yields valuable information which helps determine the mechanism of circulatory failure. Based on a pathophysiological approach integrated in the clinical context, CCE helps distinguish between persistent hypovolemia, left or right ventricular failure, tamponade, or any other source of central circulation impairment (Fig. 1). Its additional diagnostic capacity is particularly relevant in the setting of complicated open-heart surgery and for the management of patients supported with circulatory assistance where the therapeutic impact is high [10].

CCE provides accurate measurement of cardiac output [11]. Accordingly, CCE is used serially to assess the effects of passive leg raise or fluid challenge on left ventricular stroke volume [6], and its tolerance as reflected by induced changes in cardiac filling pressures [8]. When compared to other (“blind”) hemodynamic monitoring systems, CCE provides clinically relevant additional information. In 37 of 137 ventilated patients (27%) who were hemodynamically assessed for septic shock using both CCE and transpulmonary thermodilution, independent interpretation of hemodynamic profiles led to discrepant results and a source of discordance was identified by echocardiography in 43% of them [12]. Indeed, numerous hemodynamic burden or acute cardiac/great vessel conditions can only be accurately identified using CCE, including acute cor pulmonale [8, 9], dynamic left ventricular obstruction [13], massive valvular regurgitation or severe valvular stenosis, central anatomical shunts, proximal pulmonary embolism or aortic dissection [12].

Continuous improvements in electronics has enabled the emergence of miniaturized ultrasound systems and probes. Real-time three-dimensional echocardiography and new quantitative approaches for myocardial function based on speckle tracking promise to further improve the diagnostic capacity of CCE [14]. In patients with septic shock, global left ventricular longitudinal peak systolic strain measured using speckle tracking appears more sensitive than left ventricular ejection fraction to identify septic cardiomyopathy, since it is impaired in a large proportion of patients with preserved ejection fraction and it decreases before ejection fraction in the subset of patients who exhibit delayed left ventricular systolic dysfunction [15]. Whether this new index of left ventricular systolic function is prognostic remains to be established.

Overall, CCE offers a wide range of modalities and hemodynamic parameters to best help the front-line intensivist in adjusting acute therapy individually in patients with cardiopulmonary compromise, irrespective of their level of competence (basic, advanced, or expert). Recently proposed echocardiography-based management strategies and newly available technologies need further validation in large cohorts of critically ill patients. The best has yet to come.

## Abbreviation

CCE: critical care echocardiography
